# Brooklyn Dental Association

**Published:** 1864-12

**Authors:** 


### BROOKLYN DENTAL ASSOCIATION.

*October* 5, 1864.

SUBJECT:—"The comparative value of Wood's Metal as a filling for Teeth."

DR. ATKINSON stated that the component parts of Wood's Metal were bismuth, lead, cadmium and tin. He was not in the habit of using much of any metal but gold, and of all the substitutes he knew of, this he thought was the best. It sometimes, when it comes in contact with other metals, produces galvanic action, and occasions a metallic taste for a time. When well put in it serves its purpose perfectly. There are various forms of it, varying in toughness and fusibility; the objection to its use in the case of children is on account of the heat. It should be put in the cavity in very small pieces at a time. It is the best thing for very old teeth, and equally easy to work as amalgam.

DR. ROWE spoke of a case in his own mouth, where a plug of Wood's metal came in contact with a gold plug, producing an uncomfortable degree of galvanic action, accompanied with a metallic taste. He gave it as his opinion that the metal oxydizes, and softens the tooth substance, producing a yellow deposit in teeth which had been for some time filled with it, which deposit he supposed to be the oxyde of cadmium.

DR. MARVIN had filled a large cavity on the buccal side of a lower molar, extending to the gum, with Wood's metal. After the lapse of some weeks, on examining the same tooth, he found a crevice between the plug and the enamel into which the point of an instrument could be inserted, the edges of the enamel being disintegrated. He removed the filling and replugged the tooth, and after nine months he found his work in good order. He considered that more care was necessary in using this material than gold; as the filling is very apt to harden over and leave cavities underneath. The danger arises from the fact that, being a cheap filling it is likely to be inserted too hastily. He uses much smaller instruments than those offered for sale with the metal, and takes very little material at a time. His judgment and experience were very much in its favor as a better substitute for gold than any thing else.

Dr. W. H. Allen had used Wood's metal very little; he liked it pretty well. He could fill most teeth with gold that he could fill with this metal. He finds it painful in teeth where the pulp is not dead. One patient declared he wanted no more of that red hot stuff in his mouth.

Dr. C. P. Fitch said, the best filling he knew of was gold; he had always questioned the use of any base metal in the teeth: he had not come to a definite opinion on the article in question. Some patients complain very much of the heat; the temperature at which the metal becomes plastic being 163°. He finds that very small quantities must be used; if there is any difficult point that must be filled first. It requires just as nice manipulation in many cases as gold, and its success depends on the care with which it is introduced. He had never seen any of the yellow oxyde of cadmium in teeth filled with this metal. The yellow substance spoken of is found in teeth filled with gold.

Dr. Abbott finds it very difficult to fill a tooth with Wood's metal; it always wears away fast if on a grinding surface. He would prefer using gold, in any case; and does not think it equal to a good amalgam plug.

Dr. Clowes had used it in two or three cases, but the patient found it like going through Tophet; and he found it the hardest kind of a filling to put in. He has a good deal more liking for amalgam than for this. He considered the best plug one of adhesive gold malleted in, and where a gold plug was not practicable, he used one of amalgam. He had worked for many years to obtain a good amalgam plug, and he felt that he had; he stood up for amalgam, and would risk his reputation on it. His amalgam plug was tough, took a high polish, would not discolor the tooth, and so far was it from shrinking that it really expanded a little, just enough to make a very tight plug.

Dr. Fitch wanted to raise his voice against amalgam fillings. They were the dirtiest, vilest things that ever were put into the teeth. They will shrink however perfectly they are put in, and they will discolor the teeth however much you may wash them. The best amalgam plug is made of Townsend's amalgam, rubbed in a mortar with common salt, washed with water, and then pressed in chamois leather by a vice or tweezers; about 45 per cent. of the mass thus prepared is mercury.

Dr. Crowell suggested that so much candy would be of just as much use as so much salt.

Dr. Clowes stated that in all his practice he had never found any thing so offensive as an old spongy gold plug. Here was the quintessence of nastiness, the sum of all villanies. He concluded by recommending for discussion at the next meeting—"Amalgam Plugs."

